# The Surgical Treatment of Carbuncles: A Tale of Two Techniques

**DOI:** 10.5812/ircmj.2992

**Published:** 2013-04-05

**Authors:** Tan Guan Hee, Bong Jan Jin

**Affiliations:** 1 Department of General Surgery, University of Kebangsaan Malaysia, Medical Centre, Kuala Lumpur, Malaysia

**Keywords:** Surgery, Carbuncle, Debridement

## Abstract

The treatment of carbuncles is early administration of antibiotics and surgery. The commonest surgical approaches are saucerization, and incision and drainage (I&D). Although these two techniques are vastly different, there is a lack of evidence to determine which one produces a better outcome. Three cases of carbuncles are presented to illustrate the contrasting surgical techniques and their results. Three consecutive patients who presented to this hospital with carbuncles were treated with either saucerization or I&D. They were followed up for 8 weeks to assess their outcome. One patient had saucerization while two other patients underwent I&D of their carbuncles. Saucerization produced the shortest length of hospital stay. I&D resulted in earlier wound healing. A randomized controlled is needed to determine the best surgical approach for the treatment of carbuncles.

## 1. Introduction

Carbuncles are commonly associated with diabetic patients (1, 2). The treatment typically involves early administration of antibiotics and surgery (1). Opinions on the surgical treatment are divided between saucerization, and simple incision and drainage (I&D) (1, 3, 4). Despite this, there are no studies published in the English language over the last four decades that address the surgical outcome of these contrasting techniques. This case series illustrate the difference of these two surgical approaches and their result.

## 2. Case Reports

### 2.1. Case 1

**Figure 1.**
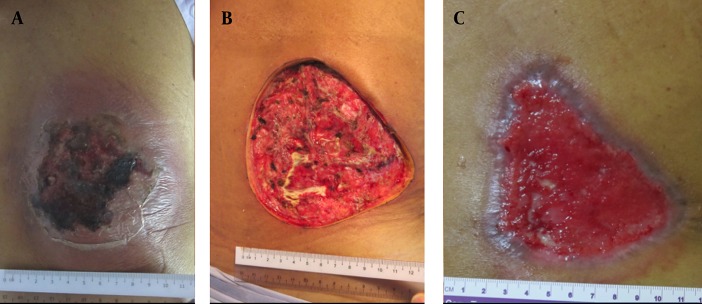
A) A Carbuncle with a Necrotic Centre and Surrounding Cellulitis. B) The Post-Operative Effect of Saucerisation. C) The Saucerized Wound Still Required Further Wound Care at 8-Weeks Post-Surgery

A 71-year-old man developed a painful carbuncle on his back 4 days prior to admission. He had diabetes mellitus, which was treated with oral hypoglycaemic medications. On arrival to the hospital, he was afebrile but had a white cell count of 21.4 x 10^9^/L. The carbuncle measured 10cm x 12cm (Figure 1 A). Saucerization of the carbuncle was performed (Figure 1 B). He was discharged on post-operative day-1 with oral co-amoxiclav for 1-week. The wound was first dressed daily and then every other day when it became cleaner. It continued to heal satisfactorily at 8-weeks after the surgery but required further follow-up for wound assessment (Figure 1 C). Pus culture from the wound grew Staphylococcus aureus. The patient had not been readmitted for wound complications or sepsis.

### 2.2. Case 2

**Figure 2.**
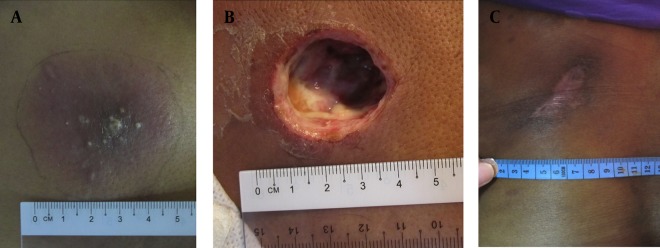
A) A Typical Appearance of a Carbuncle with Multiple Openings That Discharge Pus. B) Incision and Drainage Preserves the Surrounding Inflamed Tissue That is Later Treated with Antibiotics. C) A Healed Wound 8-Weeks after Incision and Drainage of the Carbuncle

A 47-year-old woman complained of a painful carbuncle over her left scapula for 1-week. She had a history of diabetes mellitus, which was poorly controlled. On initial presentation, she was afebrile but had a white cell count of 15.8 x 10^9^/L. Her blood glucose was 15.5mmol/L and required insulin infusion. The carbuncle was 4cm x 5cm in size (Figure 2 A). She had I&D of the carbuncle and was given co-amoxiclav for one week (Figure 2 B). She was discharged 6 days after the operation. Her wound was first cleaned every day, and then every 2 to 3-days. It healed 6 weeks after her surgery. Pus culture from the wound grew Staphylococcus aureus. There was no recurrent sepsis or readmissions after the drainage.

### 2.3. Case 3

A 47-year-old woman presented with 5 days’ history of a carbuncle on her back. She was a diabetic patient on treatment with subcutaneous insulin injections. She had a temperature of 37º C and white cell count of 24.8 x 10^9^/L. The carbuncle measured 8cm x 10cm. She underwent I&D and was treated with a week’s course of co-amoxiclav. Her recovery from surgery was uneventful and was discharged on day-5 post-operatively. The wound was initially dressed daily and then every other day when it got better. It was completely healed by about 8 weeks post-operation (Figure 2 C). The culture from the carbuncle yielded Staphylococcus aureus. She did not require readmissions during this period of follow-up.

## 3. Discussion

A carbuncle is an aggregation of multiple furuncles that form an inflammatory mass. The infected necrotic centre is walled off by a pseudocapsule. This mass typically drains onto the skin surface via several openings (3). There is usually a rim of cellulitis and inflammation around the central necrosis. This condition is commonly associated with diabetes mellitus, as demonstrated by the three cases shown here. The patient may present with sepsis that require early antibiotics administration and urgent drainage of the infection (3). Opinions on the surgical treatment of this age-old condition are divided. One group of surgeons believes that carbuncles should be widely excised in a technique called saucerisation (1, 4). This includes excision of the necrotic center and its surrounding cellulitis. The excision is deemed adequate when the limits of the surgery are healthy and completely un-inflamed. Antibiotics may not be required after saucerization. Case 1 illustrates clearly the effect of saucerization. Note that the rim of cellulitis around the center of necrosis seen in Figure 1 A was completely excised and not seen in Figure 1 B. This technique results in a large wound, which is dressed and allowed to heal by secondary intention. Occasionally, a very large wound would be closed with skin graft (1). Some might even require musculocutaneous flap or graft to cover the defect. Another group of surgeons treat carbuncles by I&D, and debridement of only the necrotic centre (3, 4). The surrounding inflamed tissue is not excised but is instead treated with a course of antibiotics. The resulting wound is smaller in this case. Similarly, it is dressed until it heals by secondary intention. This technique rarely requires grafting for wound cover because it heals fairly quickly. Cases 2 and 3 demonstrate the postoperative wound of this less radical technique. The surgical incision allowed the pus and slough to drain. The necrotic center was debrided while the surrounding cellulitis was not excised (Figure 2 B). I&D allowed the wounds to heal more quickly. Both the patients’ wounds were completely epithelialized by 8-weeks post-I&D (Figure 2 C). In comparison, the saucerized wound needed dressings for more than 8-weeks (Figure 1 C). From anecdotal comments of senior surgeons who advocate saucerization for carbuncles, it seems that it is a technique that is older than they can remember. They were taught to excise all the infected tissue, including the surrounding cellulitis in order to control the sepsis. Perhaps that was necessary in the era before effective antibiotics are widely available. However, we are now able to treat skin infections very effectively with various types of antibiotics (5). This allows a less mutilating surgery to be performed for this benign condition. There was no difference in the duration of antibiotics use in this case series. They all received a week’s course of co-amoxiclav. Interestingly, saucerisation resulted in the shortest post-operative hospital stay. All the patients did not have any further sepsis or readmission after their operation (Table 1). This case series highlight two very different surgical techniques in the treatment of carbuncles and their result. Despite this, there are no publications in the English language that address the outcome of these surgical techniques in the last 40 years. It needs to be emphasized here that this case series by no means establishes the superiority of either treatment. However, it does provide a basis to conduct a full scale randomized controlled trial to determine the best surgical approach for this condition. A randomized controlled trial is needed to answer this dilemma, which seems to have been overlooked by generations of surgeons.

**Table 1.** Summary of the Management and Outcome of the Cases

	Case 1	Case 2	Case 3
**Surgical Technique**	Saucerization	Incision and drainage	Incision and drainage
**Size of carbuncle, cm**	10 x 12	4 x 5	8 x 10
**White cell count on admission (x 10^9^/L)**	21.4	15.8	24.8
**Duration of antibiotics (days)**	7	7	7
**Post-operative length of stay (days)**	1	6	5
**Wound status at 8 weeks post-surgery**	Not healed	Healed	Healed
**Readmission/recurrent sepsis**	No	No	No
